# Nanoscale Engineering of Cobalt–Gallium Co-Doped Ferrites: A Strategy to Enhance High-Frequency Theranostic Magnetic Materials

**DOI:** 10.1021/acsanm.5c02139

**Published:** 2025-07-02

**Authors:** Itziar Galarreta-Rodríguez, Deborah Liguori, Eneko Garaio, Beatrice Muzzi, Laura Cervera-Gabalda, Juan Rubio-Zuazo, Guilherme Gomide, Jerome Depeyrot, Alberto López-Ortega

**Affiliations:** † Spanish CRG Beamline at the European Synchrotron (ESRF), B.P. 220, Grenoble F-38043, France; ‡ Instituto de Ciencia de Materiales de Madrid, ICMM-CSIC, Madrid 28049, Spain; § Center for Cooperative Research in Biomaterials (CIC biomaGUNE), Basque Research and Technology Alliance (BRTA), Donostia-San Sebastián 20014, Spain; ∥ Departamento de Ciencias, Universidad Pública de Navarra, Pamplona E-31006, Spain; ⊥ Institute for Advanced Materials and Mathematics (INAMAT^2^), 16756 Universidad Pública de Navarra, Pamplona E-31006, Spain; # 201843 ICCOM-CNR, Sesto Fiorentino (FI) I-50019, Italy; ∇ Dept. of Chemistry “U. Schiff”, University of Florence and INSTM, Sesto Fiorentino (FI) I-50019, Italy; ○ Grupo de Fluidos Complexos, Instituto de Física, 28127 Universidade de Brasília, 70919-970 Brasília, Brazil

**Keywords:** magnetism, nanoparticles, codoping, ferrites, magnetic hyperthermia, high-frequency

## Abstract

The nanoscale engineering of doped iron oxide magnetic nanoparticles has attracted significant interest in recent years for high-frequency theragnostic applications, where simultaneous diagnosis and therapy are required. In particular, their ability to generate localized heating under alternating magnetic fields makes them ideal candidates for magnetic hyperthermia, a noninvasive cancer treatment technique. However, understanding the complex interplay between multiple dopant cations and their impact on dynamic magnetic behavior remains a significant challenge. In this work, we present a comprehensive study on how two differently marked cations (Co^2+^ and Ga^3+^) can modify both the magnetic properties of these nanoparticles and their efficiency in heat generation under alternating magnetic fields. To this end, a series of nanoparticles with the formula Co_*x*_Ga_0.15_Fe_2.85‑*x*_O_4_ (0 < *x* < 0.3) was prepared via thermal decomposition, enabling the production of monodisperse nanocrystals with high crystallinity and precise stoichiometric control. Their exhaustive structural and magnetic characterization confirmed site-selective incorporation of Ga^3+^ into tetrahedral sites and Co^2+^ into octahedral sites. Increasing the cobalt content within the gallium-doped framework leads to enhanced magnetocrystalline anisotropy and higher saturation magnetization, both crucial parameters for efficient heat dissipation in magnetic hyperthermia. The study further demonstrates that the dynamic magnetic response of these nanostructures is strongly influenced by the interplay between doping composition, anisotropy, and the amplitude of the applied magnetic field. These findings highlight the effectiveness of nanoscale codoping strategies in fine-tuning magnetic behavior and optimizing the performance of spinel ferrite nanoparticles for advanced biomedical and technological applications, particularly high-frequency magnetic hyperthermia.

## Graphical abstract

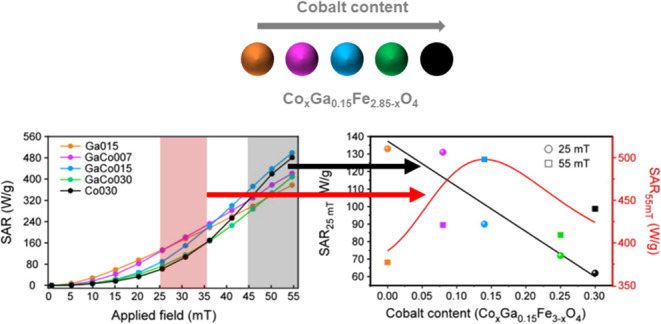

## 1 Introduction

Over the past few decades, spinel ferrite nanoparticles (NPs) have emerged as a versatile class of nanomaterials, renowned for their tunable magnetic properties and robust chemical stability. Owing to their small size and large surface-to-volume ratio, these materials exhibit size-dependent behavior that can be finely controlled at the nanoscale. Their ability to be precisely engineered at the nanoscale has enabled their deployment across a broad spectrum of applications, from energy storage and catalysis to advanced biomedical applications.^1–4^ Among these, their implementation in theragnostic nanomedicine has attracted growing attention.

In the biomedical field magnetic NPs have been employed for various applications^5^ such as magnetic separation, magnetic resonance imaging, drug delivery, and magnetic hyperthermia (MH).^3,6–9^ MH is a promising therapy that utilizes the heating capabilities of magnetic NPs under an alternating (AC) magnetic field. During this process, the NPs absorb energy from the externally applied AC magnetic field and convert it into heat.^10^ In particular, MH is emerging as a clinically relevant technique for localized cancer treatment, where precise thermal control is critical to maximize efficacy while minimizing off-target damage.^11^

In the scope of MH, the specific absorption rate (SAR) of the NPs is a key factor, which is defined as the amount of power converted into heat per mass of nanoparticles. The SAR can be obtained from the area of the magnetization vs applied field curves by means of eq 1 ^12^

1 $$SAR=-f\oint_{cycle}M_{t}dH_{app}$$

where *f* and *H*_app_ are the frequency and intensity of the externally applied magnetic field and *M*_t_ is the dynamic magnetization of the NPs (in mass magnetization units). Initially, the hysteresis area depends on the saturation magnetization and magnetic anisotropy of the NPs, both static magnetic properties. However, the dynamic magnetization *M*_t_ is strongly influenced by Néel and Brownian relaxation processes. As a relaxation process, they have a characteristic relaxation time, τ, which is the time it takes for the dynamic magnetization to return to the thermal equilibrium. Néel relaxation process (τ_N_) involves the reversal of the magnetization vector inside the NPs, while Brownian relaxation process (τ_B_) involves the physical rotation of the particles. In NPs that can rotate freely in the medium, both mechanisms modulate the effective relaxation time (τ_eff_), whereas in nonrotatable particles the τ_eff_ is practically equal to Néel time (τ_eff ≈_ τ_N_).^13^ In addition, τ_N_ depends on the externally applied field: it decreases with field amplitude.^14^ In conclusion, the efficiency of the NPs as heat agents depends not only on their static magnetic properties, like saturation magnetization and magnetic anisotropy or environmental factors such as viscosity and temperature^15^ but also on the applied magnetic field *H*_app_ amplitude, which plays an important and more intricate role.

In addition, superparamagnetic iron NPs, such as maghemite (γ-Fe_2_O_3_) and magnetite (Fe_3_O_4_), are widely studied for biomedical applications due to their biocompatibility and low cost. However, ongoing research seeks to optimize their magnetic properties to enhance their heating capabilities and reduce the required NP dosage for safe and effective treatment. Alternative strategies have been explored to improve their performance. These include investigating metal-based systems, doped-ferrite nanoparticles, and the development of core–shell heterostructures to optimize their magnetic and thermal properties.^16–18^ In this field, doped ferrites have attracted considerable interest due to their tunable properties, wide range of applications, and the ease with which they can be doped with elements other than iron.^4,7,19–25^ By selectively incorporating trivalent and divalent metal ions into the tetrahedral (Td) and octahedral (Oh) sites, the spinel structure can be engineered to exhibit customized magnetic and structural characteristics that meet specific requirements.

This tunability is demonstrated by the contrasting effects observed with different dopants. For example, cobalt-doped ferrites have been extensively studied because divalent cobalt ions in Oh sites occupy a high-spin state with an unquenched orbital moment, resulting in remarkably hard magnetic properties.^26^ Their applications span from the biomedical field,^27^ where a high MH response is crucial, to the development of new permanent magnets.^21^ Conversely, doping strategies aimed at reducing magnetic hardness have also been explored using elements such as manganese, and others.^28^ In addition to reducing anisotropy, certain applications that not require minimal losses also seek to increase only the material’s overall magnetization. To that end, introducing diamagnetic cations into a ferrimagnetic spinel can unbalance the sublattice moments between octahedral and tetrahedral sites, effectively boosting net magnetization. Examples include Zn, Mg or Ga,^7–29^ with Ga^3+^ being especially suitable because its ionic radius in tetrahedral coordination closely matches that of Fe^3+^, allowing uniform incorporation without significant lattice strain. The inclusion of gallium not only significantly modifies the magnetic properties of the structure but also renders these nanostructured systems highly promising for MRI by enabling dual contrast behavior.^7^ One known issue is the potential toxicity of Co^2+^ cations; however, studies have shown this can be mitigated by adjusting the Co/Fe ratio and/or applying appropriate surface coatings.^30–33^ In contrast, Ga^3+^-doped systems exhibit even lower intrinsic toxicity, since gallium compounds are already FDA-approved for medical imaging applications.^34^

Despite these advances, codoped systems that strategically combine multiple dopants remain underexplored, particularly in terms of their dynamic behavior under high-frequency AC fields. Building on these insights, the present study investigates the synergistic effects of combining Ga^3+^ and Co^2+^ dopants within a single spinel structure. The objective is to enhance the material’s magnetic hardness while maintaining a high overall magnetization. This dual objective is especially relevant for MH, where both high anisotropy and strong magnetization are necessary to achieve effective heating under clinical field conditions. To achieve this, we systematically vary the cobalt content in a spinel already doped with a fixed amount of gallium, examining the resulting changes in both structural and magnetic properties. Additionally, dynamic magnetic behavior is evaluated to determine the optimal doping strategy for magnetic hyperthermia applications. The present results highlight that, in this specific system, the optimal doping strategy depends not only on intrinsic magnetic properties, such as saturation magnetization and effective anisotropy, but also on the amplitude of the externally AC applied magnetic field, which influences the effective relaxation time and, consequently, the magnetic response. The present work thus offers a nanoscale engineering approach to the design of efficient heat-generating materials for use in high-frequency magnetic hyperthermia.

## 2 Materials and Methods

### 2.1 Materials

Cobalt­(II) acetylacetonate (Co­(acac)_2_, 97%), gallium­(III) acetylacetonate (Ga­(acac)_3_, 99.99%), iron­(III) acetylacetonate (Fe­(acac)_3_, 99.99%), oleic acid (OA, 90%), oleylamine (ONH_2_, 70%), 1,2-hexadecanediol (HDD, 90%) and benzyl ether (Bz_2_O, 98%) were purchased from Merck KGaA (Sigma-Aldrich). Ethanol was obtained from Panreac S.A. The solvents were used as received without further purification.

### 2.2 Synthesis of Gallium-Doped Cobalt Ferrites

Co_*x*_Ga_0.15_Fe_2.85‑*x*_O_4_ NPs were synthesized following a slightly modified procedure previously reported for the synthesis of iron-based cubic spinel (M_*x*_Fe_3‑*x*_O_4_) particles.^35^ In a regular synthesis, a mixture of *x* mmol Co­(acac)_2_, 0.1 mmol Ga­(acac)_3_, 2 – *x* mmol Fe­(acac)_3_ and 8 mmol HDD where added to a 100 mL three-neck flask containing 4 mmol OA, 4 mmol ONH_2_ and 25 mL Bz_2_O. The system was mechanically stirred and placed under nitrogen atmosphere (see Table 1). For the first 30 min, the reaction mixture was left unheated to evacuate any residual oxygen and water from the flask. The mixture was then heated to 200 °C at a rate of 4 °C min^–1^ and held at that temperature for 30 min to initiate nucleation. Subsequently, the temperature was increased to 300 °C at a rate of 2.2 °C min^–1^ and maintained at reflux for 90 min during the growth phase, through which the color of the reaction mixture changed from reddish brown to black. The solution was then cooled to room temperature (RT), and the NPs were precipitated by centrifugation (60 min, 10,000 rpm) using ethanol. The nanoparticles were redispersed in chloroform and precipitated with ethanol twice, followed by a final resuspension in chloroform. This process yielded hydrophobic dispersions of OA-coated Co_*x*_Ga_0.15_Fe_2,85‑*x*_O_4_ nanoparticles with nominal compositions of *x* = 0.08, 0.15, 0.25, which were stored in the refrigerator. Moreover, pure cobalt doped (Co_0.30_Fe_2.70_O_4_) and gallium doped (Ga_0.15_Fe_2.85_O_4_) ferrites were also synthesized following the previous described methodology to use as reference materials.

**Table 1:** Summary of the Synthetic Parameters Used during the Nanoparticle’s Preparation and Theoretical Stoichiometry

theoretical stoichiometry	Co (acac)_2_ (mmol)	Ga (acac)_3_ (mmol)	Fe (acac)_3_ (mmol)	OA (mmol)	ONH_2_ (mmol)	HDD (mmol)	Bz_2_O (ml)
Ga_0.15_Fe_2.85_O4	-	0.10	1.90	4	4	8	25
Co_0.07_Ga_0.15_Fe_2.78_O_4_	0.02	0.10	1.88	4	4	8	25
Co_0.15_Ga_0.15_Fe_2.70_O_4_	0.05	0.10	1.85	4	4	8	25
Co_0.30_Ga_0.15_Fe_2.60_O_4_	0.10	0.10	1.80	4	4	8	25
Co_0.30_Fe_2.70_O_4_	0.20	-	1.80	4	4	8	25

### 2.3 Physical, Structural and Magnetic Characterization

The structural characterization of the NPs was investigated by X-ray powder diffraction (XRD) using a Bruker New D8 ADVANCE ECO diffractometer equipped with a Cu Kα (λ = 1.5406 Å) radiation source and operated at 40 kV and 25 mA. Diffraction patterns were collected in the 2θ range 20–70°, with a step size of 0.02° and scan step speed of 0.2 s. Obtained patterns were analyzed by the Rietveld profile refinement method using MAUD program.^36^ Microstrain and crystal size were calculated using the isotropic size-strain model.

Fe K-edge X-ray absorption spectroscopy (XAS) experiments were performed at the Spanish BM25 beamline of the ESRF (Grenoble, France). X-ray absorption near edge structure (XANES) spectra were recorded at RT in fluorescence mode. The energy of the absorption spectra was calibrated by measuring the XANES of a Fe metal foil. For the measurements, homogeneous layers of the powdered samples were prepared by spreading fine powders of the material onto iron-free adhesive carbon tape. XANES spectra were recorded on the different samples and on a series of iron oxide references: Fe_3_O_4_ (magnetite) and α-Fe_2_O_3_ (hematite). The absorption spectra were analyzed according to standard procedures by using the ATHENA program package^37,38^

The relative cation concentrations of cobalt, gallium and iron in the NPs were measured with an Inductively coupled plasma atomic emission spectroscopy (ICP-AES) by Agilent, Vista-MPX model. Approximately 1.5 mg of the powder was dissolved in 0.6 mL freshly prepared *aqua regia* (1:3 nitric acid:hydrochloric acid). This solution was then diluted with 25 mL of Milli-Q water and used for ICP measurements.

The morphology and size distribution of the NPs were analyzed by Transmission electron microscopy (TEM) using a TALOS F200X G2 (Thermo-Fisher Scientific). This microscope is equipped with a high-brightness Field Emission Gun (X-FEG, 80–200 keV) and four in-column SDD Super-X detectors for Energy-dispersive X-ray spectroscopy (EDXS), which was used to determine the elemental composition of the NPs. For each sample, the mean diameter and size distribution of the NPs were obtained by statistical analysis of over 100 NPs using the ImageJ software.^39^

The magnetic properties of the NPs were measured on tightly randomly packed powder samples using a Vibrating sample magnetometer (VSM) with 1.5 T maximum field and a Cryogenic S700X-R SQUID Magnetometer up to 7 T equipped with a cryostat that can measure from 2 to 400 K. The magnetization versus applied field measurements, M­(H), were recorded both at RT (300 K) and at low temperature (5 K) after 5 T field-cooled (FC) process. The magnetization versus temperature, M­(T), measurements were carried out in zero-FC (ZFC) and 0.5 mT FC conditions. In addition, the dynamic magnetization of the NPs under alternating magnetic field measurements was also measured by means of an AC Magnetometer (ACM) with a coupled optic fiber temperature sensor (Neoptix).^40^ The specific absorption rate (SAR) was obtained from the area of the M­(H) curves by means of eq 1.

## 3 Results and Discussion

### 3.1 Structural and Morphological Characterization of Nanoparticles

A series of cobalt, gallium, and cobalt/gallium-doped ferrite NPs were synthesized following a slightly modified procedure previously reported for the synthesis of iron-based cubic spinel particles through thermal decomposition of organometallic compounds in a high-boiling solvent with OA and ONH_2_ as stabilizing surfactants (see Scheme 1).^7^ The desired amounts of cobalt, gallium and iron precursors were adjusted during the synthetic preparation to obtain the specific stoichiometry described in Table 1. This approach produced stable dispersions of Co_0.3_Fe_2.7_O_4_ (Sample **Co030**), Ga_0.15_Fe_2.85_O_4_ (Sample **Ga015**), and Co_*x*_Ga_0.15_Fe_3‑*x*_O_4_, where *x*, the number of cobalt cations, ranges from 0.07 to 0.30 (Samples **GaCo007**, **GaCo015** and **GaCo030**, respectively). The actual cobalt, gallium, and iron contents were determined by ICP-AES spectroscopy, revealing that cobalt, gallium and iron percentages closely matching the theoretical values used in the synthesis. Table 2 includes the calculated chemical formulas based on this analysis. Figure 1a–e shows some representative bright-field, low-magnification TEM images of the as-prepared doped ferrite NPs, along with their corresponding particle size histograms. The morphological analysis reveals that most nanoparticles exhibit shapes consistent with a spherical or cuboctahedral morphology. Nevertheless, some variability is observed, with smaller particles tending toward a more spheroidal shape, while larger ones display triangular or rectangular profiles. The average particle sizes across the sample series range from 11 to 14 nm, with a relative size dispersion of approximately 20%, indicating that the system cannot be considered monodisperse (Table 2). All the size histograms obtained from TEM micrographs by evaluating the particle diameter, *D*_TEM_, are well fitted by a log-normal distribution, indicating a single size population with a narrow diameter distribution (deviation < 30%).^41^ These similar characteristics across all samples are expected, as identical synthesis conditions were employed, particularly through the control of the nucleation/growth process and the use of equal OA/ONH_2_ and metal/surfactants ratio.^21^ In addition, high-angle annular dark field (HAADF) imaging and EDX elemental mapping were performed on sample **GaCo030** and is depicted in Figure 1f. The analysis shows that all the cations, i.e., cobalt, gallium, and iron, are uniformly distributed throughout the studied NPs with no evidence of atomic segregation. This confirms the homogeneity of the as-synthesized particles, and the elemental ratio obtained by EDX (Co_0.28_Ga_0.15_Fe_2.57_O_4_) is consistent with that one previously determined by ICP analys.

**Figure 1:**
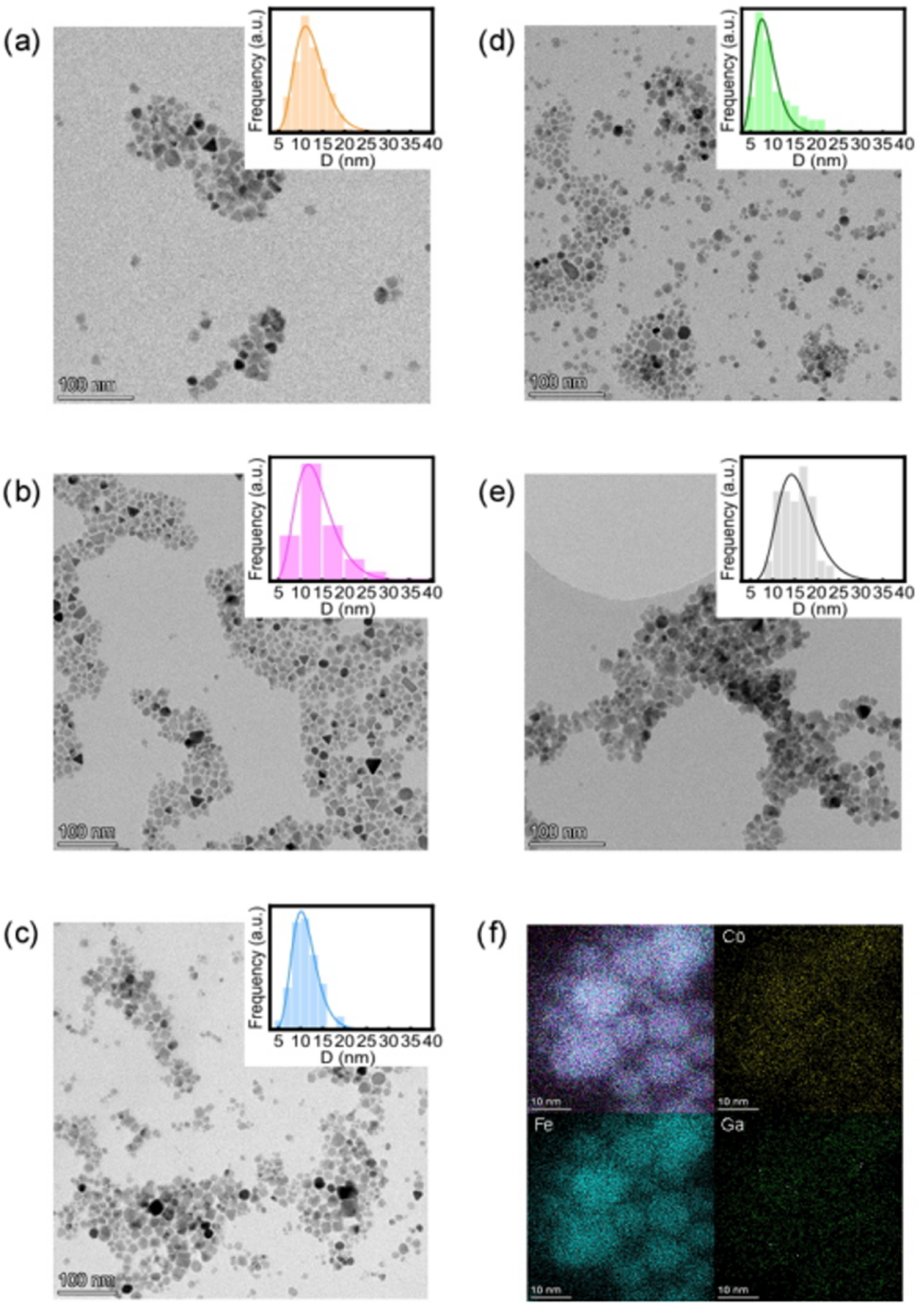
Low resolution TEM images and particle size histograms for samples (a) **Ga015**, (b) **GaCo007**, (c) **GaCo015**, (d) **GaCo030** and (e) **Co030** (Scale bars correspond to 100 nm). (f) HAADF image and EDX mapping for sample **GaCo030** showing the elemental composition of the particle, where Fe, Co and Ga are depicted in light blue, yellow and green, respectively (Scale bars correspond to 10 nm).

**Scheme 1:**
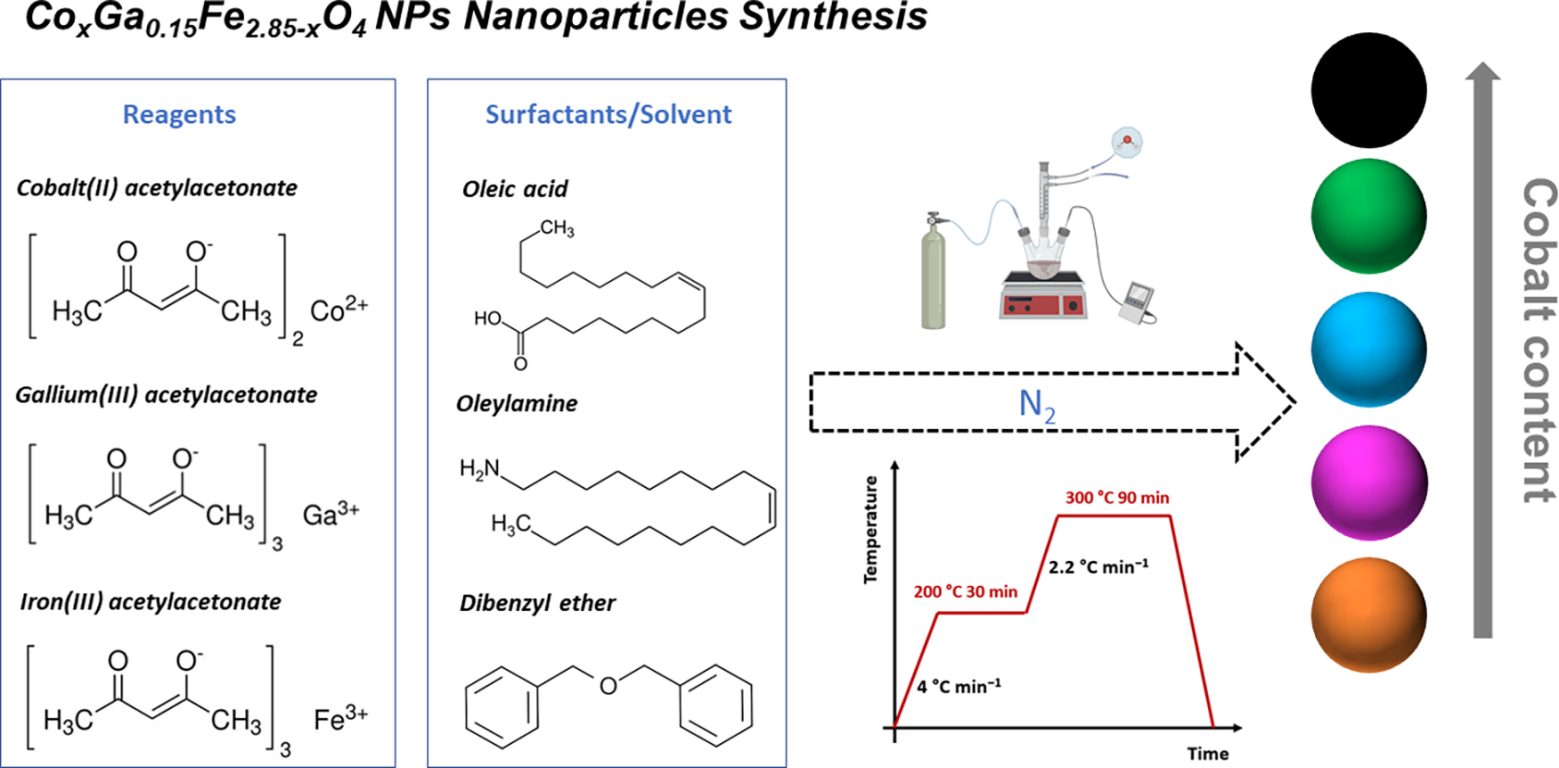
Schematic Representation of the Co_*x*_Ga_0.15_Fe_2.85‑*x*_O_4_ (0 < *x* < 0.3) Nanoparticle Synthesis

**Table 2:** Chemical and Structural Data of the As-Synthesized Doped Ferrite NPs^a^

sample	theoretical stoichiometry	actual stoichiometry	*D*_TEM_ (nm)	*D*_DRX_ (nm)	*a* (nm)	ε (arb. units)
**Ga015**	Ga_0.15_Fe_2.85_O_4_	Ga_0.18_Fe_2.82_O_4_	12(3)	13.8(5)	0.8375(1)	0.00162
**GaCo007**	Co_0.07_Ga_0.15_Fe_2.78_O_4_	Co_0.08_Ga_0.18_Fe_2.74_O_4_	14(4)	14.6(2)	0.8381(1)	0.00175
**GaCo015**	Co_0.15_Ga_0.15_Fe_2.70_O_4_	Co_0.14_Ga_0.16_Fe_2.70_O_4_	11(3)	16.0(3)	0.8384(1)	0.00272
**GaCo030**	Co_0.30_Ga_0.15_Fe_2.60_O_4_	Co_0.25_Ga_0.18_Fe_2.56_O_4_	11(3)	14.5(6)	0.8389(1)	0.00291
**Co030**	Co_0.30_Fe_2.70_O_4_	Co_0.30_Fe_2.70_O_4_	13(3)	15.5(5)	0.8391(1)	0.00321

a Theoretical and actual stoichiometry were calculated from synthetic parameters and ICP analysis, respectively. The *D*_TEM_ average particle diameter was obtained from TEM measurements. The *D*_DRX_ average crystal size, the cell parameter (a) and the microstrain (ε) were calculated from Rietveld refinement of the XRD patterns. Note that the values in parentheses refer to the standard deviations, and the error for ε has been estimated of the 10% of the calculated value.

The structural characterization of the as-synthesized particles was carried out using powder XRD analysis. The XRD patterns shown in Figure 2a display broad and poorly defined peaks because of the small crystallite domain size and the presence of organic matter coating the NPs. All the diffraction peaks can be indexed as the cubic structure of spinel oxides (Fd3*m*, JPCDS no. 89–0691), confirming the crystalline nature of the samples with no evidence of secondary phases. The crystal size, *D*_DRX_, as evaluated from the diffraction patterns, is consistent with that obtained from TEM images, indicating the growth of single-crystal NPs and their high crystallinity. Interestingly, the *D*_DRX_ values are very similar across the entire series of NPs, indicating that they do not depend on the different composition. It should be noted, however, that minor differences between XRD- and TEM-derived sizes can occur in polydisperse systems, due to the different averaging nature of the two techniques. In addition, the cell parameter (*a*) and microstrain (ε) were determined and are reported in Table 2 and Figure 2b.

**Figure 2:**
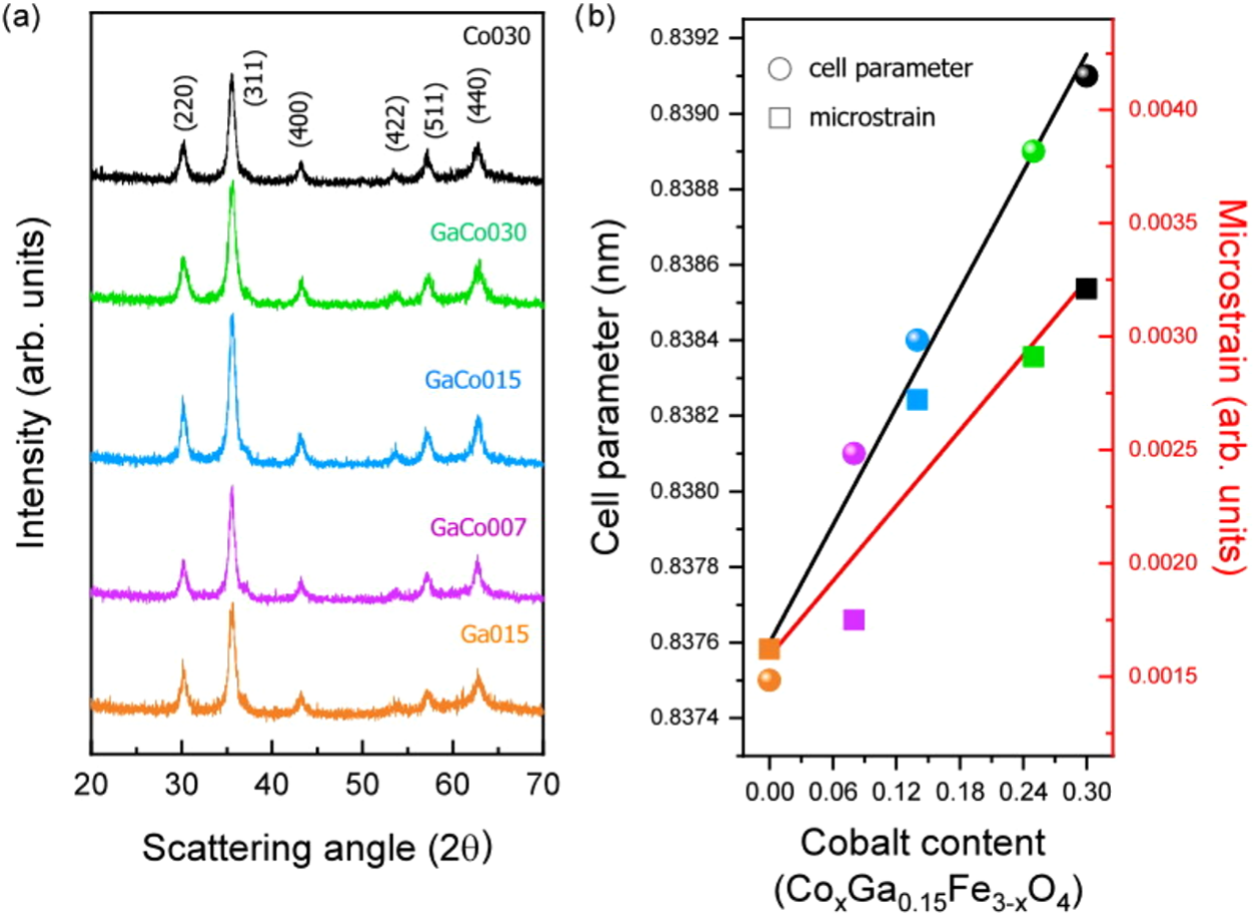
(a) XRD patterns for the series of doped ferrite NPs and (b) cell parameter (left scale and circular symbols) and microstrain (right scale and square symbols) dependence on cobalt content. The black and red lines serve as visual guides for the cell parameter and microstrain linear dependence, respectively.

Both the lattice parameter and the microstrain show a clear linear dependence on the cobalt content. As shown in Figure 2b, the cell parameter for the **Ga015** sample is slightly below the value expected for pure magnetite (0.8380 nm).^42^ However, as the cobalt content increases, the lattice parameter progressively rises, eventually reaching values close to that of cobalt ferrite (0.8389 nm).^21^ As previously reported for gallium-doped nanoparticles with doping concentrations below 10%, Ga^3+^ ions tend to occupy Td positions of the spinel structure (Ga^3+^: 0.61 Å in Td). At higher gallium contents, however, their occupancy in Oh positions increase (Ga^3+^: 0.76 Å in Oh).^7,43^ Thus, as gallium is incorporated into the spinel structure, the Fe^2+^: Fe^3+^ ratio evolves from 1:2 (in magnetite) toward 1:1 (as in GaFe_2_O_4_), and given the larger ionic radius of Fe^3+^ (Fe^3+^: 0.78 Å in Oh, 0.63 Å in Td), this redistribution leads to a reduction in the cell parameter compared to pure magnetite. Conversely, when cobalt is introduced, the lattice parameter tends to increase. This trend is somewhat counterintuitive, as Co^2+^ (0.89 Å in Oh) is expected to replace Fe^2+^ (0.92 Å in Oh), which would theoretically result in a slight decrease in the lattice parameter based on ionic size alone.^43–45^ However, this apparent contradiction can be explained by a more complex structural evolution. First, Co^2+^ may not only substitute Fe^2+^ but also partially replace Fe^3+^ in octahedral positions, leading to an average increase in ionic size at Oh sites. To preserve electroneutrality, such substitution may also require slight adjustments in the oxygen stoichiometry.^46,47^ Second, gallium could occupy both Td and Oh sites depending on doping levels and local structural conditions. The incorporation of Co^2+^, which strongly prefers Oh sites, may displace Ga^3+^ ions into Td positions. Since Ga^3+^ is smaller than Co^2+^ and Fe^3+^ in Oh coordination, this cation redistribution would further contribute to the observed expansion of the lattice.

In addition, cobalt incorporation is associated with a significant increase in microstrain, likely caused by local structural distortions due to ionic size mismatch and lattice relaxation effects. This increase in microstrain may also play a role in the observed expansion of the cell parameter.^48,49^

To gather more detailed structural information about the as-synthesized doped ferrite NPs, XANES spectra at the Fe K-edge were recorded for samples **GaCo007**, **GaCo015**, **GaCo030** and **Ga015**, along with two references: magnetite (Fe_3_O_4_) and hematite (α-Fe_2_O_3_), see Figure 3a. In particular, the XANES spectra provide valuable complementary information on the metal oxidation states and cation site symmetries. Based on the Fe K-edge position, the studied samples fall between the two references, i.e., 7121.9 (5) eV and 7123.9 (5) eV for magnetite and hematite, respectively, (see Figure 3) consistent with iron oxides composed of a combination of Fe^2+^ (Oh) and Fe^3+^ (Td, and Oh).

**Figure 3:**
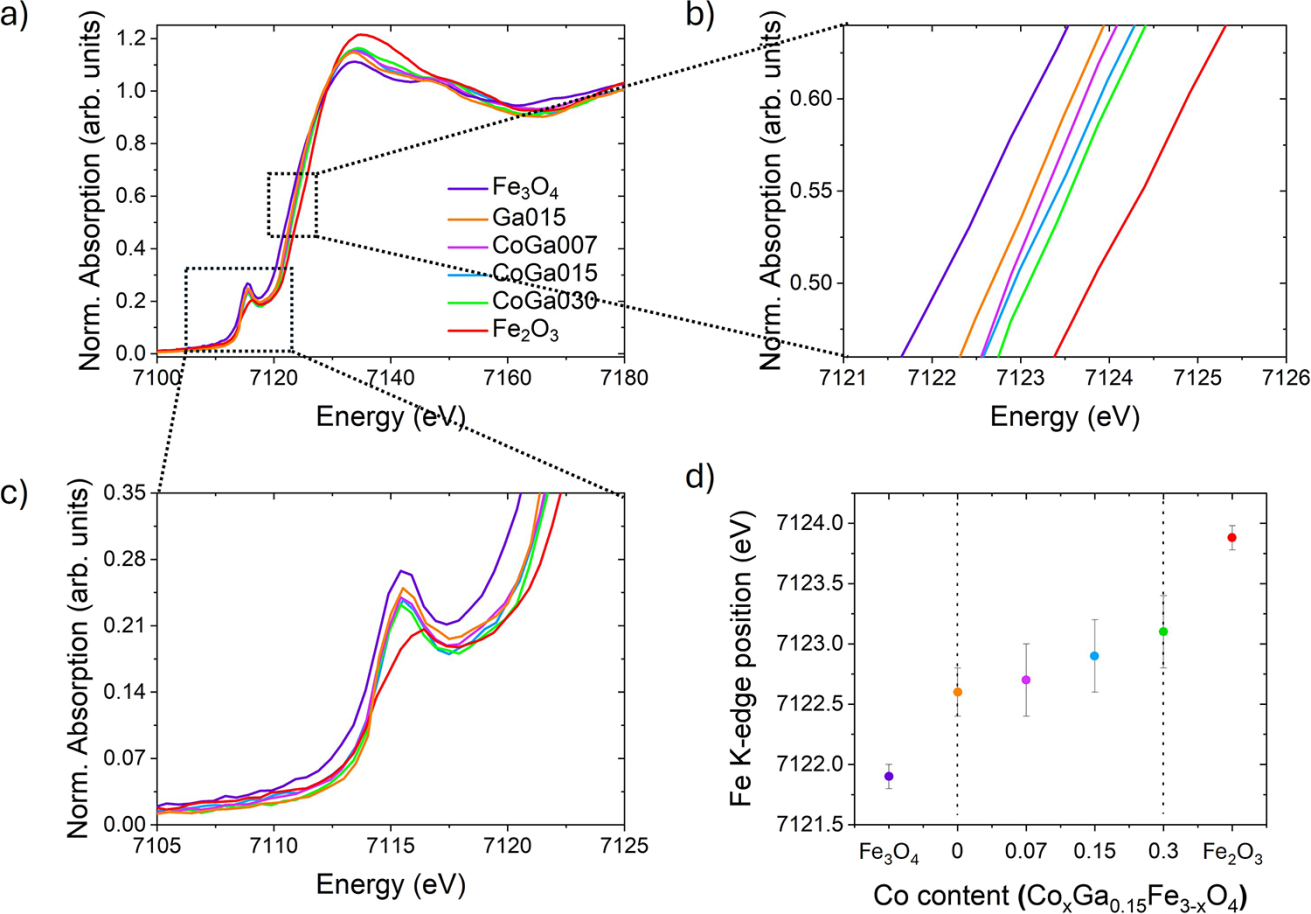
(a) Normalized XANES spectra for samples **GaCo007**, **GaCo015**, **GaCo030 and Ga015** and two references: magnetite (Fe_3_O_4_) and hematite (α-Fe_2_O_3_). (b) Inset of the edge region of the XANES spectra. (c) Inset of the pre-edge region of XANES spectra. (d) Dependence of the Fe K-edge position in function of the cobalt content.

This fact indicates that all structures have a similar iron cation distribution, i.e., Fe^2+^ and Fe^3+^, corroborating the diffraction data that show a cell parameter value very close to that of magnetite. As shown in Figure 3b, the absorption edge shifts progressively to higher energies upon the introduction of Ga^3+^ alone, and even more so when both Ga^3+^ and Co^2+^ cations are incorporated. This can be explained by the fact that the inclusion of the gallium occupies only Td sites, thus not displacing or eliminating Fe^2+^. In contrast, when cobalt is included in this structure, we observe a shift of the Fe K-edge toward higher energies, i.e., approaching the hematite reference. In this case, the shift indicates that the oxidation state of iron is moving toward Fe^3+^, suggesting that Co^2+^ cations are being incorporated into the structure by substituting Fe^2+^ ions in Oh sites. As a result, the doped spinel structure becomes more similar to that of the hematite reference, which consists only Fe^3+^ cations . This fact is further corroborated by considering that the shift of the Fe K-edge toward higher energies increases monotonically with the amount of cobalt in the crystalline structure, as is depicted in Figure 3d, similarly to what was observed for the cell parameter (Figure 2b). The increase in the iron oxidation state is also reflected in the enhanced white line intensity, which becomes more pronounced with higher Co incorporation.

In addition, this trend can also be observed in the shoulder located below the edge position (Figure 3c). The pre-edge peak arises from electronic transitions between the 1s core state and the 3d orbitals, which are typically dipole-forbidden by selection rules. The intensity and position of this peak offer valuable insights into the local symmetry and electronic environment of the absorbing atom.^50,51^ While the magnetite reference exhibits a sharper and more intense pre-edge peak; the fully trivalent hematite spectrum presents a broader and less intense signal. Thus, taking into account the position of the pre-edge peak in our samples and comparing them with those of the reference materials, it can be confirmed that both Fe^2+^ and Fe^3+^ are present in the samples.

Additionally, previous studies have indicated that the intensity of the pre-edge feature tends to increase with the deviation from a centrosymmetric coordination environment.^50^ As a result, the pre-edge absorption intensity follows the order of octahedral, five-coordinate, and tetrahedral sites, with increasing intensity corresponding to the increasing distortion from centrosymmetry.^51^ Specifically, in spinel structures, the pre-edge peak intensity is typically higher in tetrahedral or distorted octahedral environments, while it tends to decrease in undistorted octahedral coordination. Interestingly, the whole series of NPs studied show similar spectra to that of magnetite, but the intensity of this pre-edge peak decreases monotonically with the dopant’s concentration in the sample. For the Ga015 sample, the observed decrease in pre-edge peak intensity can be ascribed to the preferential incorporation of Ga^3+^ into tetrahedral coordination sites, which in turn promotes a higher occupancy of Fe in Oh environments, as previously discussed. However, upon cobalt incorporation, the pre-edge peak intensity continues to decrease, as cobalt substitutes some of the iron atoms, particularly in Oh sites, thereby modifying the crystal field and reducing the centrosymmetry of the local coordination environment.

### 3.2 Static Magnetic Characterization

In order to evaluate the magnetic performance of the cobalt inclusion into the gallium-doped NPs, an in-depth analysis of their static and dynamic magnetic properties was performed using magnetometric techniques. In particular, magnetization measurements were conducted with respect to the magnetic field, M­(H), and temperature, M­(T). In Figure 4, the ZFC-FC M­(T) curves for the series of NPs are presented.

**Figure 4:**
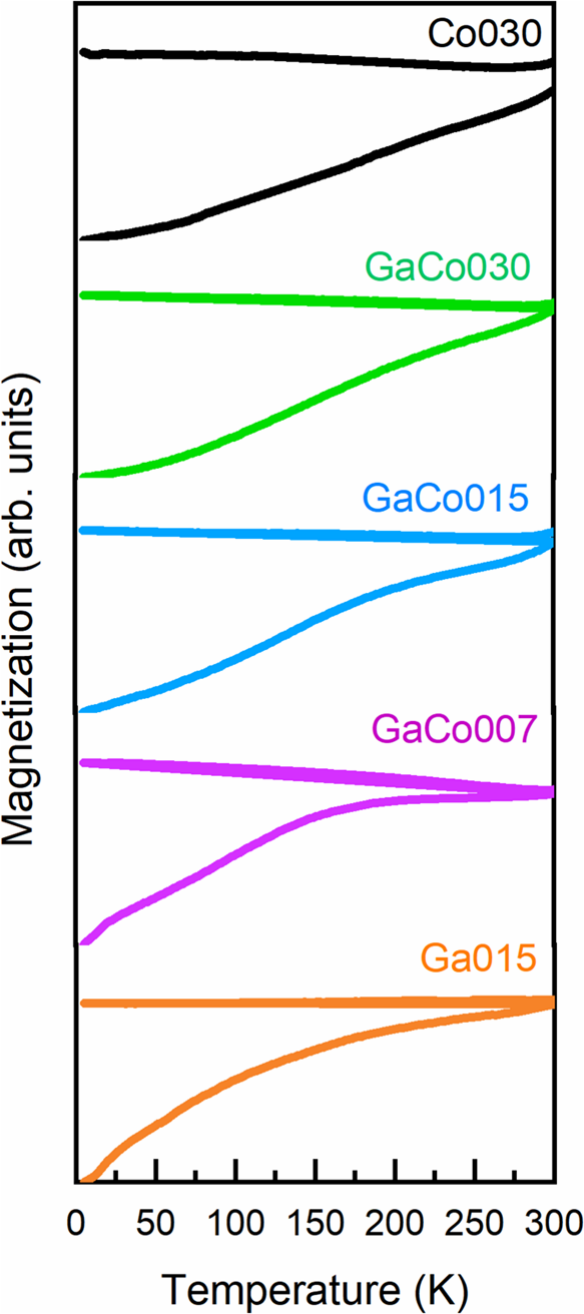
ZFC-FC M­(T) curves for all the series of samples.

In the ZFC curve, no observable maximum (*T*_MAX_) associated with the blocking temperature appears within the tested temperature range. Moreover, the divergence point between the ZFC and FC curves (referred to as irreversibility) is not observed even at 300 K in most cases. This suggests that the nanoparticles remain in a blocked state at least up to this temperature. Apart from this, the absence of a *T*_MAX_ in the ZFC data, along with the nearly constant FC magnetization below the irreversibility point, implies strong interparticle dipolar interactions within the samples. These interactions result in a collective magnetic behavior rather than isolated responses from individual NPs, which can lead to magnetic frustration and give rise to the nonequilibrium characteristics observed in magnetic glassy states.^52,53^ Although the nanoparticles are coated with oleic acid, the typical interparticle spacing in dried powders is small, and magnetic nanoparticles of this size may still exhibit dipolar collective behavior when closely packed. Interestingly, this strong collective behavior observed in the entire series leads to a significant increase in the magnetic relaxation time, well beyond what would be expected solely from the nanoparticle anisotropy energy barrier (KV, where K and V are the magnetocrystalline anisotropy and particle volume, respectively).^54^ For instance, in the case of pure Fe_3_O_4_ NPs, a blocking temperature above 300 K would typically require particle diameters that are 5–10 nm larger than those of the particles in our samples.^55,56^ These results are supported by the M­(H) curves measured at RT, shown in Figure 5a and summarized in Table 3. All samples exhibit open hysteresis loops with a coercive field (H_C_) and remanence (M_R_) that remain nonzero, both increasing as cobalt doping in the structure rises (see inset in Figure 5a). However, it is worth noting that the **Ga015** sample exhibits values close to zero, indicating that it is near its superparamagnetic transition and has lower magnetocrystalline anisotropy compared to the cobalt-doped samples.

**Figure 5:**
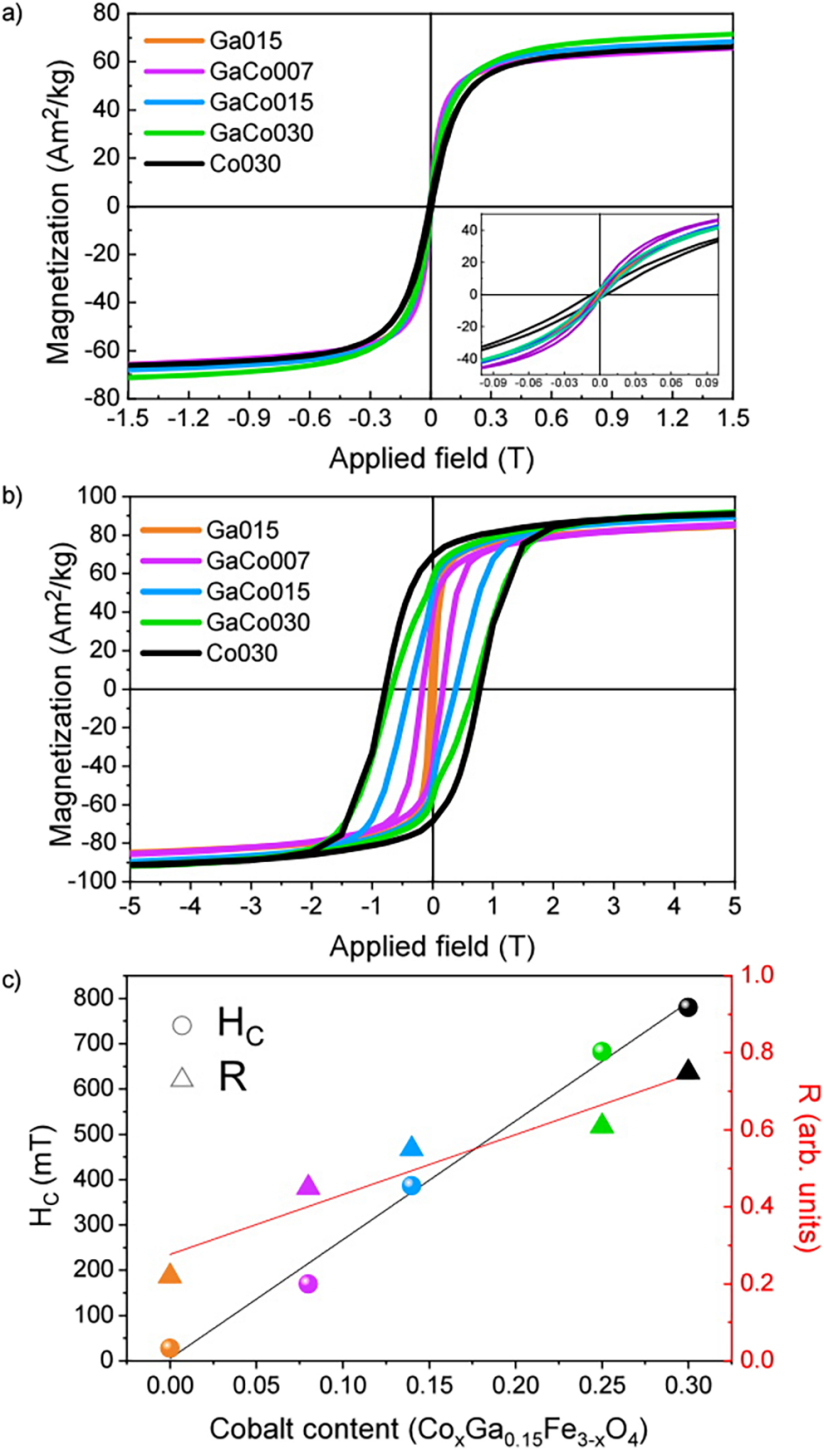
(a) RT and (b) 5 K M­(H) curves for all the series of samples. The inset in Figure 4a depicts zoom for the low field region. (c) H_C_ (left scale and circular symbols) and M_R_ (right scale and triangular symbols) dependence in function of the cobalt content for the 5 K M­(H) curves. The black and red lines serve as visual guides for the H_C_ and M_R_ linear dependence, respectively.

**Table 3:** Analysis of the Magnetic Properties Obtained from Static at RT and 5 K and Dynamic at RT Hysteresis Loop Measurements: Magnetization at High Fields (M_1.5T_ and M_5T_, for Static at RT and 5 K Respectively), Remanence Magnetization (M_R_), Reduced Remanence (R = M_R_/M_1.5T_ or M_5T_), Coercive Field (H_C_) and the Specific Absorption Rate at 25 mT (SAR_25 mT_) and 55 mT (SAR_55 mT_)

	RT			5 K				AC RT			
sample	M_1.5T_ (Am^2^/kg)	M_R_ (Am^2^/kg)	H_C_ (mT)	M_5T_ (Am^2^/kg)	M_R_ (Am^2^/kg)	R	H_C_ (mT)	M_55mT_ (Am^2^/kg)	H_C_ (mT)	SAR_25mT_ (W/g)	SAR_55mT_ (W/g)
**Ga015**	65.9	1	0.070	84.5	18.6	0.22	27.9	37.5	9.4	133	377
**GaCo007**	65.8	2.9	0.220	85.2	38.3	0.45	169.7	34.8	12.1	131	421
**GaCo015**	68.4	3.2	0.340	89.3	49.1	0.55	386.4	31.1	16.5	90	498
**GaCo030**	71.4	3.3	0.350	91.3	55.7	0.61	682.3	24.2	17.2	72	409
**Co030**	77.2	3.1	0.620	91.0	68.3	0.75	780.0	23.2	20.8	62	440

To gain a deeper insight into the impact of cobalt inclusion, we measured the magnetization response as a function of the applied field at low temperatures. As shown in Figure 5b, all M­(H) curves exhibit the characteristic hysteretic behavior typical of ferro- or ferrimagnetic particles without any horizontal loop shift (i.e., associated with exchange-bias coupling) thus, corroborating the absence of cationic segregation within the particles.^57^ However, we can observe that as the cobalt content increases, the M­(H) loops become progressively broader. This trend is clearly reflected in Figure 5c, where both the H_C_ and reduced remanence (R = M_R_/M_5T_) increase linearly with the cobalt content in the doped ferrite. Initially, the **Ga015** sample exhibits an H_C_ of 27.9 mT, a value consistent with the low magnetocrystalline anisotropy expected for these types of doped ferrite materials.^7^ Indeed, it must be stressed that the Ga^3+^ ion has a negligible magnetic moment; however, its inclusion can reduce the tetrahedral-octahedral exchange coupling, thereby softening the magnetic character of the NPs.^58^ Introducing cobalt causes H_C_ to increase substantially, ranging from 169.7 mT to 682.3 mT for samples **GaCo007** and **GaCo030**, respectively, reaching its maximum of 780.0 mT for the pure cobalt-doped, **Co030** sample. Meanwhile, although R also increase linearly, the values for the cobalt/gallium-doped NPs remains well below those expected for cubic magnetocrystalline anisotropy (i.e., 0.82–0.86), which suggests a shift in magnetic anisotropy symmetry from uniaxial to cubic as the cobalt content increases.^59^ This monotonic rise in both H_C_ and R is attributed to the enhancement of magnetocrystalline anisotropy in the nanoparticles, driven by the substitution of Fe^2+^ by Co^2+^ in the Oh sites, which presents stronger spin–orbit coupling due to the specific crystal field splitting that Co^2+^ suffers in Oh environments. Additionally, the magnetization at high fields, both at low (M_5T_) and RT (M_1.5T_), appears dependent on the cobalt doping level (see Table 3). This outcome can be attributed to both cobalt and gallium doping within the ferrimagnetic iron oxide structure and the reduced nature of the material. Specifically, cobalt substitution is expected to slightly reduce the magnetization value, since Co^2+^ (magnetic moment of 3 μB) replaces Fe^2+^ (4 μB) in octahedral sites. The magnetization values for the **Co030** sample are consistent with those previously reported in the literature.^21,60,61^ In contrast, gallium inclusion can increase the high-field magnetization when Ga^3+^ (nonmagnetic) substitutes Fe^3+^ in tetrahedral sites, thereby reducing the opposing sublattice moment.^7^ However, the lower-than-expected magnetization observed in the Ga-only doped sample suggests that Ga^3+^ is likely distributed between both Td and Oh sites, which results in an overall dilution of the magnetic lattice and limits the enhancement effect. Interestingly, when cobalt is introduced into the system, the high-field magnetization increases with cobalt content. This observation, which at first seems counterintuitive considering the lower moment of Co^2+^ compared to Fe^2+^, can be explained by a redistribution mechanism: the presence of Co^2+^ in Oh sites forces Ga^3+^ to preferentially occupy Td positions. As a result, the dilution of the Oh sublattice is minimized, and the imbalance between magnetic sublattices increases, leading to a net rise in magnetization.

Finally, we would like to highlight two additional observations: the relatively high magnetization of the pure cobalt ferrite sample within the series, and the unexpectedly low value of the Ga-only doped sample. Although a full explanation requires further investigation, we propose that the lower crystallinity in the Ga-containing samples could play a role. This may be linked to the higher thermal stability of Ga­(acac)_3_ compared to Fe­(acac)_3_, which could lead to incomplete decomposition during synthesis and potentially introduce structural defects that reduce the magnetic performance.^62,63^

### 3.3 Dynamic Magnetic Characterization

Figure 6a depicts the dynamic magnetization M_t_(H) curves, also known AC loops, recorded at 310 kHz and maximum field amplitude of 55 mT. Interestingly, at this frequency, the curves exhibit a clear open hysteretic behavior, demonstrating their capability to generate heat under alternating magnetic fields. However, what is most noteworthy is that the loop shape appears to be strongly related to the structure’s doping level. Given that the AC loop shape is determined by the effective relaxation time (τ_eff_) of the dynamic magnetization,^17^ we can confirm that this magnetization is also strongly influenced by the cobalt content. Experimentally, we can observe that while sample **Ga015** shows lower H_C_ values and higher magnetization than the rest of the series, increasing the cobalt content causes H_C_ to rise and the overall magnetization to decrease (see Figure 6a and Table 3), a behavior which is clearly related with the length of τ_eff_.

**Figure 6:**
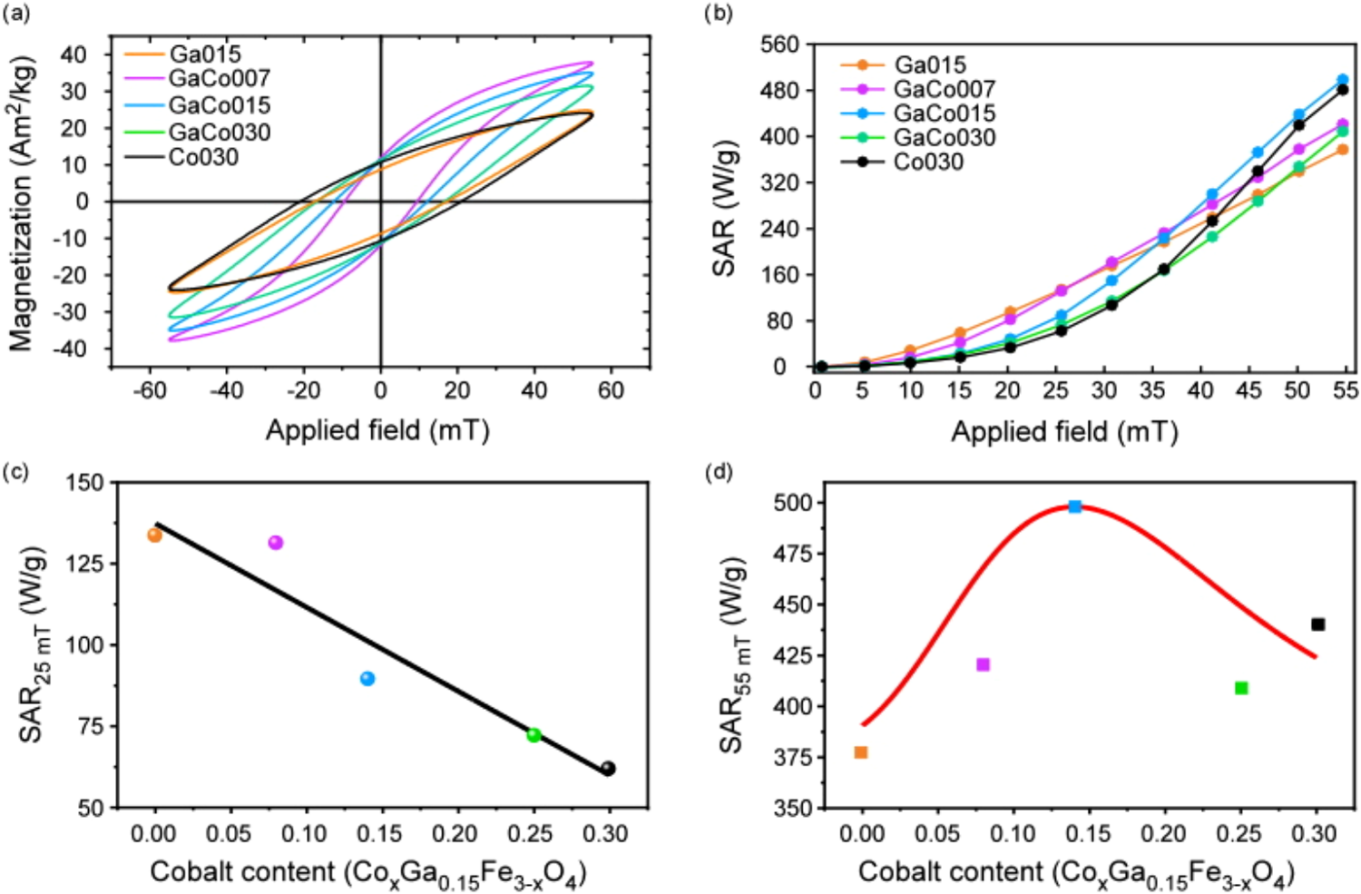
(a) RT AC loops recorded at 310 kHz with a maximum amplitude field of 55 mT, (b) SAR in function of the field amplitude and SAR dependence in function of the cobalt content at (c) 25 and (d) 55 mT. Note that the (c, d) axes present different scales for SAR and the black and red lines serve as visual guides for the 25 and 55 mT dependence, respectively.

First, the H_C_ of the AC loops increase with longer τ_eff_ times because the magnetization do not have enough time to follow the alternating magnetic field. Consequently, short τ_eff_ values yield narrow dynamic magnetization curves. In addition, when τ_eff_ is long, the magnetization cannot reach equilibrium values within the limited time available, which lowers the measured dynamic magnetization, especially at low field amplitudes. In contrast, at higher amplitudes, this reduction in the magnitude of the dynamic magnetization tends to vanish because a stronger field effectively shortens the energy required to overcome the anisotropy barrier and flip the magnetization, resulting in a reduction of τ_eff_.^14^ Therefore, the dynamic magnetization analysis confirms that sample **Ga015** exhibits the shortest effective relaxation time among the entire series. Given that the NPs are not dispersed in a fluid, the effective relaxation time closely approximates the Néel time (τ_eff_ ∼ τ_N_). Consequently, a shorter τ_eff_ corresponds to a lower effective anisotropy, which is consistent with the static magnetic properties.

Finally, Figure 6b and Table 3 present the SAR measured at 310 kHz as a function of the applied field. Because the SAR is proportional to the area of the AC hysteresis loop, the effective relaxation mechanism governing the nanoparticles’ dynamic properties directly influences the SAR values and, consequently, their heating efficiency. The SAR reaches a maximum value when the τ_eff_ equals the AC magnetic field oscillation period, *P*. Hence, if τ_eff_ < *P*, the hysteresis loop area increases as τ_eff_ grows. Conversely, if τ_eff_ > *P*, the loop area decreases with increasing relaxation time.^64^ At field amplitudes below 30 mT, the SAR is higher in samples with shorter τ_eff_, thereby decreasing from **Ga015** to **Co030** samples (see Figure 6c). However, at amplitudes above 40 mT, the situation reverses, and the SAR follows a nonmonotonic trend with a maximum SAR for the intermediate **GaCo015** sample (see Figure 6d). This nonlinear behavior arises because a larger field amplitude effectively reduces the effective relaxation time: once τ_eff_ becomes shorter than P, the trend fully reverse and the SAR decrease from **Co030** to **Ga015**. Therefore, it is clear that the most efficient samples as hyperthermia agents, that is, the ones with the maximum SAR value, also strongly depend on the externally applied field amplitude, as the effective relaxation time decreases with the applied magnetic field. However, under real conditions where it is necessary to consider a safety limit for frequency and amplitude, i.e., H·*f* < 5·10^9^ Am^1–^ Hz (although the limit can be even higher for smaller application regions). We can see that for high applied magnetic field amplitudes (i.e., 55 mT), the optimal composition differs markedly from the case of lower field amplitudes (i.e., 25 mT) close to the safety limit.

## 4 Conclusions

In summary, we have demonstrated the feasibility of synthesizing nanoscale cobalt/gallium-doped spinel ferrite nanoparticles with the composition Co_*x*_Ga_0.15_Fe_2.85‑*x*_O_4_ (0 < *x* < 0.3) via a thermal decomposition method using organometallic precursors. This synthetic process enables the production of highly crystalline nanostructures with precise control over particle size and dopant concentration. The results indicate that increasing the cobalt content in a gallium-doped matrix leads to enhanced magnetocrystalline anisotropy in the series of NPs; moreover, high-field magnetization is maintained or improved in comparison with Co-ferrite NPs synthesized by similar methods.^21,60,61,65^ X-ray diffraction and XANES analyses confirmed the systematic structural changes induced by the dopants, providing direct evidence of nanoscale structural engineering. Static and dynamic magnetization measurements underscored the critical role of the dopant ratio in determining overall magnetic properties and heat generation capacity under alternating magnetic fields. Moreover, the analysis of the dynamic magnetic properties revealed not only the most efficient dopant combination for generating heat under magnetic fields but also that this heating efficiency is intimately linked to both the field amplitude and the material’s anisotropy, an essential consideration for biomedical applications. These findings validate the efficacy of nanoscale codoping strategies for fine-tuning the performance of spinel ferrite nanoparticles and highlight their potential for use in theragnostic applications, particularly magnetic hyperthermia-based cancer therapies, where both high heating efficiency and controlled magnetic behavior are crucial.
